# Supplementary material for: Cardiomyocyte Differentiation from Mouse Embryonic Stem Cells by WNT Switch Method
Source: Cells. 2024 Jan 11;13(2):132. doi: 10.3390/cells13020132 (PMC10814988; doi:10.3390/cells13020132)
Supplement: Supplementary file 1 [file cells-13-00132-s001.zip › Table S1.pdf]

Table s1: RT-qPCR primers used in study

Primer Name	*Strand*	*Sequence*
Nanog	F	ACC TGA GCT ATA AGC AGG TTA AGA C
Nanog	R	GTG CTG AGC CCT TCT GAA TCA GAC
Oct3/4	F	GTT CTC TTT GGA AAG GTG TTC AGC
Oct3/5	R	ATC TCC TGA AGG TTC TCA TTG TTG T
Brachyury	F	CTGTGACTGCCTACCAGAATGAGGAG
Brachyury	R	GGTCGTTTCTTTCTTTGGCATCAAG
Wnt3	F	CCGCTTCTGTCTAGGGTCTG
Wnt3	R	GGTAGAGAGTGCAGGCAAGG
Gata4	F	CTCTATCACAAGATGAACGGCATCAAC
Gata4	R	TCTGGCAGTTGGCACAGGAGAG
Lhx1	F	CCTGGACCGCTTTCTCTTGAA
Lhx1	R	ACCGAAACACCGGAAGAAGTC
cTnT	F	GAGGAGGTGGTGGAGGAGTA
cTnT	R	GGCTTCTTCATCAGGACCAA
Myh6	F	GATGGCACAGAAGATGCTGA
Myh6	R	CTGCCCCTTGGTGACATACT
Nkx2.5	F	CCACTCTCTGCTACCCACCT
Nkx2.5	R	CCAGGTTCAGGATGTCTTTGA
Gapdh	F	CAAAATGGTGAAGGTCGGTGTGAA
Gapdh	R	CAACAATCTCCACTTTGCCACTG
